# Characterization of the amino-terminal domain of Mx2/MxB-dependent interaction with the HIV-1 capsid

**DOI:** 10.1007/s13238-014-0113-5

**Published:** 2014-11-04

**Authors:** Jia Kong, Bo Xu, Wei Wei, Xin Wang, Wei Xie, Xiao-Fang Yu

**Affiliations:** 1 School of Life Science, Tianjin University, Tianjin, 300072 China; 2 Collaborative Innovation Center of Chemical Science and Engineering, Tianjin, 300072 China; 3 Department of Molecular Microbiology and Immunology, Johns Hopkins Bloomberg School of Public Health, Baltimore, MD 21205 USA; 4 Institute of Virology and AIDS Research, First Hospital of Jilin University, Changchun, 130061 China

**Dear Editor**,

More than 50 years have passed since the *myxovirus resistance* (*MX*) genes were first discovered and found to suppress infection with influenza viruses in mice (Lindenmann, 1962). Like most mammals, mice carry two *MX* genes, *MX1* and *MX2*, which have arisen by gene duplication; both of these genes exhibit antiviral activity against a wide range of viruses (Liu et al., 2013). Humans also have two *MX* genes, encoding the MxA and MxB proteins, which are interferon-induced, dynamin-like large molecular weight guanosine triphosphatases (GTPases). The antiviral functions of MxA have been deeply explored: MxA can protect cells from infection by multiple groups of pathogenic DNA and RNA viruses, such as influenza A virus and hepatitis B virus (Liu et al., 2013). In contrast, Mx2, although closely related to MxA (63% amino acid [aa] sequence identity), appears to have lost its antiviral function and has been recognized as playing other cellular roles, since it does not suppress the viruses tested (Melen et al., 1996).

Recently, Mx2 has been shown to serve as an inhibitor of human immunodeficiency virus type-1 (HIV-1) (Goujon et al., 2013; Kane et al., 2013; Liu et al., 2013). Mx2 restricts HIV-1 infection at a relatively late post-entry phase (Goujon et al., 2013; Kane et al., 2013) and leads to a reduced level of integrated viral DNA (Liu et al., 2013). The N-terminal 91-aa domain of Mx2 has been identified as a critical determinant of Mx2’s antiviral activity (Busnadiego et al., 2014). Interestingly, several mutations in the HIV-1 viral capsid (CA) region of Gag can overcome Mx2-mediated suppression (Goujon et al., 2013; Kane et al., 2013; Liu et al., 2013). Thus, Mx2 may bind to the HIV-1 core and inhibit the early events in HIV-1 binding, thereby restricting viral infection. However, there is still no evidence showing that Mx2 directly binds to the HIV-1 capsid. Whether capsid binding of Mx2 requires cellular co-factors and/or higher-order assemblies of CA is also unknown. In this study, we have obtained a stable Mx2 protein containing the N-terminal 91-aa domain. Furthermore, we have observed that purified Mx2 recombinant proteins bind directly to HIV-1 CA assemblies *in vitro*. The N-terminal 83-aa domain of Mx2 is apparently critical for this interaction.

Human MxA and Mx2 share a similar aa sequence and domain architecture (Liu et al., 2013; Melen et al., 1996). The crystal structure of MxA indicates that it includes a G domain that binds and hydrolyzes GTP; a hinge-like “bundle signaling element (BSE)” that connects the G domain to the elongated stalk domain; and the stalk domain, which is involved in self-assembly and oligomerization (Gao et al., 2010; Gao et al., 2011) (Fig.1A). A unique feature of Mx2 is that it exhibits a longer N-terminal domain, including an NLS (N-terminal 25 aa).

**Figure 1:**
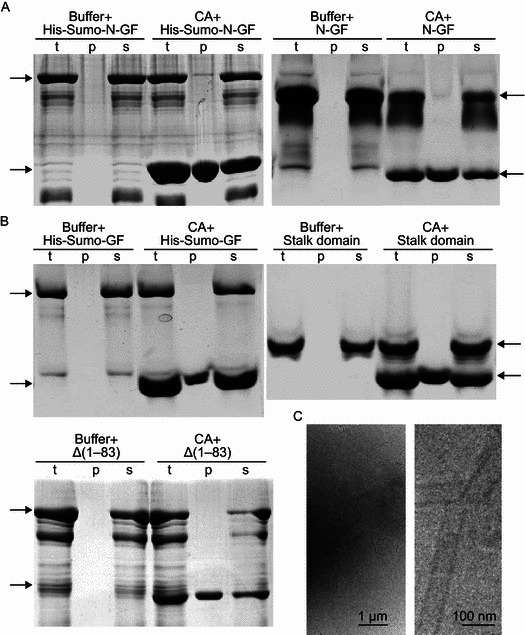
**Interactions of Mx2 variants with CA tubular assemblies**. (A) Binding of His-sumo-N-GF and N-GF to pre-assembled wild-type CA tubes. Binding reactions were analyzed by SDS-PAGE using CA tubular assemblies at 80 μmol/L or binding buffer alone, incubated with His-sumo-N-GF and N-GF (79 μmol/L). Samples of the reaction mixture before centrifugation (t), of the supernatant (s), and of the pellet (p) are detected alone. (B) Binding of Mx2 constructs to preassembled wild-type CA tubes. Binding reactions were the same as in (A). No binding of any Mx2 construct without the N-terminal 83-aa domain was found. (C) Cryo-EM images of pre-assembled wild-type CA tubes used in the Mx2-CA binding experiments

To identify the contribution of the diverse Mx2 domains to capsid binding, we generated and screened a series of deletion constructs of Mx2 with an N-terminal His-Sumo-tag (Fig. S1A) and characterized the expression, solubility, stability, and oligomerization behavior of these constructs (Table S1). We found that full-length Mx2 was difficult to obtain in *E. coli*. The expression levels of all the other deletion constructs (1–387, 1–413) were greatly reduced, but ∆1–83 exhibited a highly improved expression level (Fig. S1B), indicating that the N-terminal 83-aa domain of Mx2 may cause low expression and instability in solution (Table S1). Other deletion constructs (84–387, 84–413) without the N-terminal 83-aa domain showed high expression levels (Fig. S1B), but it exhibited poor solubility and stability (Fig. S1C), indicating that the completeness of BSE plays a significant role in maintaining the structure of the Mx2 constructs. Thus, we were unable to obtain stable constructs with the N-terminal 83-aa domain.

To overcome this problem, on the basis of previous reports (Chappie et al., 2010) and the structure of MxA (Gao et al., 2011), we engineered a minimal GTPase-BSE fusion protein (GF) that connected residues 84–413 and residues 683–715 from human Mx2 via a flexible linker (Fig. S1A). GF eluted as a monodispersed peak from a size-exclusion column and had much better solubility (>20 mg/mL) than did residues 84–387 and 84–413 (Fig. S1C). In the next phase, we added the N-terminal 83-aa domain of Mx2 to GF in order to generate an N-terminal-GTPase-BSE fusion protein (N-GF). His-Sumo-N-GF showed a highly improved expression level (>20 mg/L in *E. coli*) and solubility when compared to other deletion constructs with the N-terminal 83-aa domain (Fig. S1C). After removing the Sumo-tag, we saw a reduction in the stability of the N-GF proteins, but N-GF eluted as a monodispersed peak from a size-exclusion column (Table S1). Thus, we successfully generated a stable fusion construct of Mx2 for use in detecting further binding.

It had previously been demonstrated that assembled HIV-1 CA-NC tubular complexes can interact with full-length MX2 and its truncations containing the N-terminal 91-aa (isolated from HEK293T cell lysates) (Fricke et al., 2014). To further clarify whether Mx2 directly binds to HIV-1 CA assemblies (at 20 μmol/L) *in vitro*, we tested purified His-Sumo-N-GF and N-GF (79 µmol/L) in precipitation assays with preassembled CA tubes. The results clearly indicated that the N-GF proteins co-pellet with assembled CA, revealing that the binding process does not require other cellular co-factors (Fig. 1A); thus, established that Mx2 directly binds to HIV-1 CA tubes *in vitro*.

We performed a further dissection of the interactions between the Mx2 constructs and CA to address the contribution of the different Mx2 domains to capsid binding. Purified GF, ∆1–83, and the stalk domain (79 μmol/L) were each tested in the precipitation assay with preassembled CA tubes (20 μmol/L). Essentially no binding was observed for GF, ∆1–83, or the stalk domain under the same assay conditions (Fig. 1B). All CA tubes were prepared as previously described (Yang et al., 20122012; Hung et al., 2013) and were confirmed by cryo-electron microscopy (cryo-EM) (Fig. 1C). The findings described above indicate that Mx2-CA binding requires the N-terminal 83-aa of Mx2. The N-GF does not contain the stalk domain, but it is apparently sufficient to bind HIV-1 CA assembly. On the other hand, the stalk domain alone showed no binding to the assembled CA tubes (Fig. 1B).

HIV-1 capsid (CA) proteins can assemble into closed fullerene cones or helical tubes *in vivo* and *in vitro*. It is widely known that tripartite motif protein isoform 5 alpha (TRIM5α) is an antiviral protein that restricts infection of HIV-1 by binding to the viral capsid. The TRIM5α-CA binding interaction requires an assembled capsid lattice, since individual CA monomers do not have an appreciable affinity for TRIM5α (Stremlau et al., 2006). It is the shape of the HIV-1 capsid that is recognized by the TRIM5α protein (Yang et al., 2012). Having confirmed the binding of Mx2-CA assemblies, we wanted to investigate whether, like TRIM5α, Mx2 binds to the viral capsid by recognizing the shape of the higher-order assembled HIV-1 capsid lattice. Therefore, we over-expressed and purified individual hexamers (A14C/E45C/W184A/M185A), pentamers (A21C/E22C/W184A/M185A), and monomers of CA as previously described (Fig. 2A) (Pornillos et al., 2011), and separately examined the binding affinity between each of them and GST-N-GF by GST pull-down assays, detecting the results by SDS-PAGE. No complex formation was detected, even at very high concentrations of both proteins (Fig. 2B). This result demonstrates that Mx2 shows no detectable binding to CA hexamers, pentamers, or monomers, implying that a higher-order lattice of CA tubes is required for an efficient Mx2-CA interaction.

**Figure 2:**
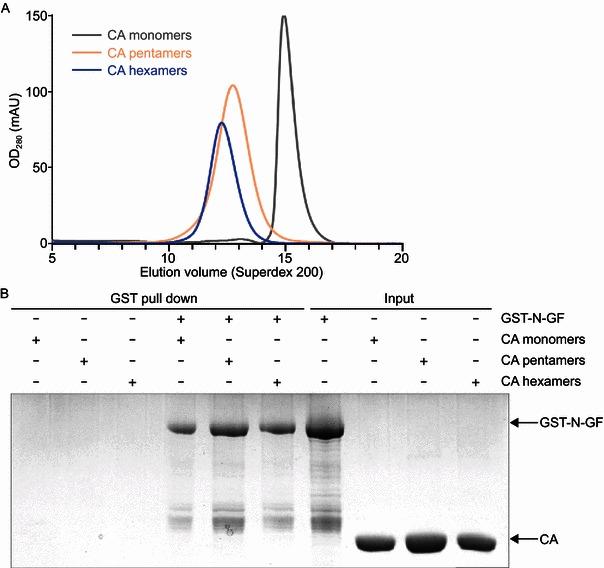
**GST Pull-down assays of GST-N-GF with CA hexamers, pentamers, and monomers**. (A) Size-exclusion chromatographic profiles of individual CA hexamers, pentamers, and monomers. (B) Pull-down assays of GST-N-GF with CA hexamers, pentamers, and monomers. CA hexamers, pentamers, and monomers, incubated with GST-N-GF-bound glutathione-sepharose beads, showed no more interaction than those incubated with glutathione-sepharose beads alone

In the current study, we have demonstrated for the first time that a direct interaction occurs between Mx2 and the HIV-1 capsid. The N-terminal region of Mx2 is critical for this interaction. We identified a fusion protein of Mx2 containing the N-terminal 83-aa and the complete BSE (N-GF), which was fundamental for the detection of Mx2-CA binding *in vitro*. The N-GF construct may also facilitate further biochemical and structural studies of Mx2. The co-pelleting of purified N-GF and CA tubes, as well as domain mapping studies, showed that Mx2 directly recognizes CA assembles through its N-terminal 83-aa domain. Based on the results of pull-down assays, Mx2-CA binding requires the higher-order assembled capsid lattice, just as TRIM5α-CA binding does.

While this work was in progress, it was reported that assembled HIV-1 CA-NC tubular complexes can co-pellet Mx2 from whole-cell lysates (Fricke et al., 2014). However, there is no evidence to show that Mx2 directly binds to CA *in vitro* (i.e., whether capsid binding of Mx2 requires co-factors in cells). The contribution of the NC domain to the binding of Mx2 to higher-order assemblies of CA-NC was also not clear. Our data argue that there is direct interaction between Mx2 and HIV-1 CA. Interestingly, several mutations in the HIV-1 viral capsid (CA) region of Gag can overcome MX2-mediated suppression (Fricke et al., 2014; Liu et al., 2013). The CA-NC tubular complexes that are formed with mutations in CA that help HIV-1 escape from Mx2 restriction exhibit weakened interactions with Mx2 (Fricke et al., 2014). Thus, Mx2 may bind to the HIV-1 core and inhibit the process of HIV-1 infection.

It has been reported that Mx2 variants ∆572–715 and ∆623–715 have lost their ability to oligomerize, and they fail to bind to CA-NC assemblies *in vitro* (Fricke et al., 2014). Our results show that although N-GF lacks the stalk domain involved in oligomerization, it still has the ability to bind CA tubes. This binding may occur because N-GF has the complete BSE, which is of great importance in maintaining normal Mx2 structure. These results suggest that although it is possible that the oligomerization of Mx2 produces a higher binding affinity for the viral capsid, oligomerization is not necessary for Mx2-CA binding. Future studies will be required to explore the oligomerization functions that are required for Mx2-mediated HIV-1 restriction (Haller et al., 2010).

## Electronic supplementary material

Below is the link to the electronic supplementary material.

Supplementary material 1 (PDF 4686 kb)
