# Supplementary material for: Characterization of the amino-terminal domain of Mx2/MxB-dependent interaction with the HIV-1 capsid
Source: Protein Cell. 2014 Nov 4;5(12):954–7. doi: 10.1007/s13238-014-0113-5 (PMC4259888; doi:10.1007/s13238-014-0113-5)
Supplement: Supplementary file 1 — Supplementary material 1 (PDF 4686 kb) [file 13238_2014_113_MOESM1_ESM.pdf]

Supplementary Materials

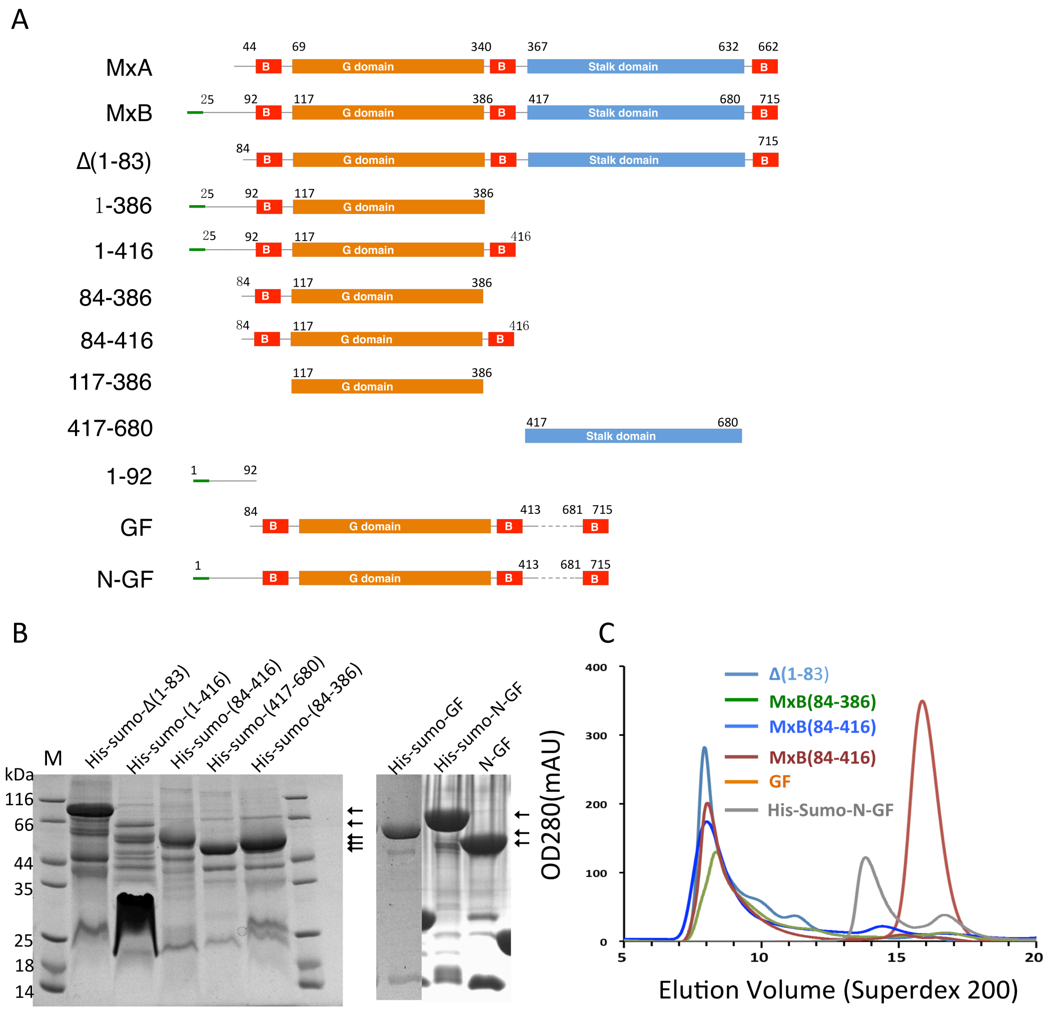

Figure S1

Architecture of the Mx2 constucts. (A) Structure-based domain representation of human Mx2 constructs. Wild-type human MxA and Mx2 are depicted in the top figure. The different Mx2 variants are shown. The numbers of the amino acid residues at the boundaries of the domains are indicated. (B) SDS-PAGE of different His-Sumo-Mx2 constructs purified using Ni-NTA columns. The N-terminal 83-aa domain causes low levels of His-Sumo-(1-416) expression and solubility. (C) Gel-filtration profiles of different Mx2 constructs. The N-terminal His-Sumo-tags have been removed. Δ1-83, 84-416, and the stalk domain (417-680), mainly in the aggregation fraction; GF, only in the monomeric fraction. (D) Gel filtration profiles of His-Sumo-N-GF and N-GF. His-Sumo-N-GF and N-GF are mostly in the monomeric fraction. The Sumo-tag improved the stability of N-GF.

Mx2 constructs	Expression[a]	Solubility[b]	Stability[c]	Oligomerization[d]
Full Length	-	ND	ND	ND
1-387	+	-	ND	ND
1-416	+	-	ND	ND
Δ(1-83)	++	++	+++	+
84-387	++	+	+	+
84-416	++	+	+	+
117-387(GTPase)	++	+	+	+
417-681(Stalk)	+++	+++	+++	+
1-83	-	ND	ND	ND
GF	+++	+++	+++	-
N-GF	++	++	++	-

Table S1 Behavior of recombinant Mx2 proteins

ND: Not determined.

[a]Expression was measured as the percentage of the expressed His-Sumo-tag-Mx2 construct in the total cell lysate. The percentage expression was determined by SDS-PAGE. "+++": indicates 100%, which corresponds to the amount of GF expression; "+": indicates ~10% of the GF expression level; "-": indicates no detectable expression.

[b]Solubility was determined by the amount of each His-sumo-Mx2 variant purified from Ni-NTA-affinity chromatography. "+++": indicates 100% of the amount of purified GF; "+": indicates ~10% of the amount of purified GF; "-": indicates no target protein was obtained.

[c]Stability was measured by the amount of target Mx2 variant in solution after removal of the Sumo-tag/GST-tag. "+++": indicates 100% of the amount of GF; "+": indicates ~10% of the amount of GF; "-": indicates no target protein was detected.

[d] Oligomerization was analyzed by size chromatography. ,"+": indicates most of the protein oligomerized, "-":indicates most of the protein was monomeric.

Materials and Methods

Expression and purification of Mx2 constructs

The fragments encoding Mx2 deletion constructs were PCR-amplified from full-length Mx2 cDNA (XM_005260983) and cloned into the modified pET-30a plasmid with a His-Sumo-tag at the N-terminus. The N-GF and GF fusion proteins were created by PCR amplification from His-sumo-Mx2 (full-length) and His-Sumo-Δ1-83 using the following 5'-phosphate primers:

5'-ggatcccaagagcagagtgagaccgctacc-3';

5'-gcttccgatgtcagccccgcaacgc-3'.

All proteins were over-expressed overnight at 18°C in *Escherichia coli* BL21 (DE3) cells. The cells were lysed using a French press in buffer containing 50 mM Tris-HCl, pH 8.0, with 300 mM NaCl, 20 mM imidazole, 1 mM PMSF, and 0.5% Triton X-100. The eluted fractions from Ni-NTA affinity chromatography (GE Healthcare) were collected and analyzed by SDS-PAGE to assess expression and solubility. The His-Sumo tag was cleaved by ULP in buffer (20 mM Tris-HCl, pH 8.0, with 300 mM NaCl and 5 mM DTT) and removed by Ni-NTA affinity chromatography. The target proteins were sequentially detected on a Superdex200 sizing column (GE Healthcare) in buffer (20 mM Tris-HCl, pH 8.0, with 150 mM NaCl and 0.5 mM TCEP).

In vitro assembly of HIV-1 CA

The expression and purification of the monomeric HIV-1_{NL4-3} CA protein have been described in detail(Hung et al., 2013). Wild-type CA proteins were assembled in vitro by overnight dialysis into assembly buffer (50 mM Tris-HCl, pH 8.0, with 1 M NaCl) containing 20 mM βME at 4°C. Final protein concentrations were about 10 mg/mL. Assembled particles were visualized by cryo-EM.

The CA mutations A14C/E45C/W184A/M185A and A21C/E22C/W184A/M185A were generated by site-directed mutagenesis and confirmed by custom sequencing analysis.

Cross-linked CA A14C/E45C/W184A/M185A hexamers and CA A21C/E22C/W184A/M185A pentamers were prepared separately as previously described (Pornillos et al., 2009; Pornillos et al., 2011). In brief, the monomers of CA mutants were purified like wild-type CA, with only the minor difference being that the buffer contained 200 mM βME. Purified CA mutants were sequentially dialyzed into assembly buffer containing 200 mM βME, assembly buffer with 0.2 mM βME, and, finally, 50 mM Tris-HCl, pH 8.0.

The CA hexamers and pentamers were then detected on a Superdex200 sizing column.

Binding of Mx2 variants to assemblies of CA

To verify binding, 20 μl of assembled CA tubes (20 μM) were incubated in vitro with 80 μl of PBS supplemented with 1M NaCl, pH 7.5, and each Mx2 variant (79 μM) at 37°C for 1 h. A fraction of this mixture was stored (total). The mixture was then centrifuged at 100,000*g* for 1 h at 4 °C. After centrifugation, the supernatant (soluble) was carefully removed, and the pellet (pellet) was resuspended in 1× SDS-PAGE loading buffer (pellet). The protein concentration was determined by SDS-PAGE.

***In vitro* GST pull-down assays**

The *in vitro* binding assays were performed according to standard procedure: GST-N-GF-bound glutathione-sepharose beads were incubated with purified CA hexamers, pentamers, and monomers in binding buffer (20 mM HEPES, pH 7.5, with 150 mM NaCl, 1

mM DTT, and 0.5% Triton X-100) for 2 h at 4°C. Protein-bound resins were washed five times using binding buffer, and the bound samples were analyzed using SDS-PAGE.

References

Hung, M., Niedziela-Majka, A., Jin, D., Wong, M., Leavitt, S., Brendza, K.M., Liu, X., and Sakowicz, R. (2013). Large-scale functional purification of recombinant HIV-1 capsid. PLoS One *8*, e58035.

Pornillos, O., Ganser-Pornillos, B.K., Kelly, B.N., Hua, Y., Whitby, F.G., Stout, C.D., Sundquist, W.I., Hill, C.P., and Yeager, M. (2009). X-Ray Structures of the Hexameric Building Block of the HIV Capsid. Cell *137*, 1282-1292.

Pornillos, O., Ganser-Pornillos, B.K., and Yeager, M. (2011). Atomic-level modelling of the HIV capsid. Nature *469*, 424-427.
